# cAMP Response Element Binding Protein1 Is Essential for Activation of Steroyl Co-Enzyme A Desaturase 1 (*Scd1*) in Mouse Lung Type II Epithelial Cells

**DOI:** 10.1371/journal.pone.0059763

**Published:** 2013-04-18

**Authors:** Nisha Antony, Jacqui R. Weir, Annie R. A. McDougall, Theo Mantamadiotis, Peter J. Meikle, Timothy J. Cole, Anthony D. Bird

**Affiliations:** 1 Department of Biochemistry and Molecular Biology, Monash University, Clayton, Victoria, Australia; 2 Baker IDI Heart and Diabetes Institute, Melbourne, Victoria; 3 The Ritchie Centre, Monash Institute of Medical Research, Monash University, Clayton, Victoria, Australia; 4 Department of Pathology, University of Melbourne, Parkville, Victoria, Australia; University of Giessen Lung Center, Germany

## Abstract

Cyclic AMP Response Element-Binding Protein 1 (*Creb1*) is a transcription factor that mediates cyclic adenosine 3′, 5′-monophosphate (cAMP) signalling in many tissues. *Creb1^−/−^* mice die at birth due to respiratory failure and previous genome-wide microarray analysis of E17.5 *Creb1^−/−^* fetal mouse lung identified important Creb1-regulated gene targets during lung development. The lipogenic enzymes stearoyl-CoA desaturase 1 (*Scd1*) and fatty acid synthase (*Fasn*) showed highly reduced gene expression in *Creb1^−/−^* lungs. We therefore hypothesized that Creb1 plays a crucial role in the transcriptional regulation of genes involved in pulmonary lipid biosynthetic pathways during lung development. In this study we confirmed that *Scd1* and *Fasn* mRNA levels were down regulated in the E17.5 *Creb1^−/−^* mouse lung while the lipogenic-associated transcription factors *SrebpF1*, *C/ebpα* and *Pparγ* were increased. *In vivo* studies using germline (*Creb1^−/−^*) and lung epithelial-specific (*Creb1^EpiΔ/Δ^*) Creb1 knockout mice showed strongly reduced Scd1, but not Fasn gene expression and protein levels in lung epithelial cells. *In vitro* studies using mouse MLE-15 epithelial cells showed that forskolin-mediated activation of Creb1 increased both *Scd1* gene expression and protein synthesis. Additionally, MLE15 cells transfected with a dominant-negative ACreb vector blocked forskolin-mediated stimulation of *Scd1* gene expression. Lipid profiling in MLE15 cells showed that dominant-negative *ACreb* suppressed forskolin-induced desaturation of ether linked lipids to produce plasmalogens, as well as levels of phosphatidylethanolamine, ceramide and lysophosphatidylcholine. Taken together these results demonstrate that *Creb1* is essential for the induction and maintenance of *Scd1* in developing fetal mouse lung epithelial cells.

## Introduction

Prematurity is a major complication for newborn infants and accounts for ∼60% of the perinatal morbidity and mortality associated with birth. This is primarily due to an incomplete development of the lung which cannot adequately fulfil the demands of oxygenation for the body. As a result, premature infants often suffer varying degrees of respiratory distress syndrome (RDS) with the severity depending on the degree of lung immaturity. A key event of late lung development is the differentiation and maturation of the type II alveolar epithelial cell (AEC) in the distal lung, which primarily functions to synthesize and secrete surfactant into the airways. Lung surfactant is composed of approximately 90% phospholipids, and 10% surfactant associated proteins [1], [2]. This complex mixture reduces the surface tension at the air-liquid interface after birth that prevents alveolar collapse and therefore allows normal lung function. To a large degree, the severity of RDS is closely associated with a profound lack of type-II AEC differentiation and deficiency of lung surfactant (reviewed in [3]). The genetic programs which drive pulmonary morphogenesis, and in particular stimulate epithelial cell differentiation and surfactant production in the lung are controlled by the actions of specific transcription factors, which regulate a complex array of gene expression networks.

Among the many transcription factors identified to have a vital role in the developing lung is the cyclic adenosine 3′,5′-monophosphate (cAMP) response element binding protein (Creb1). *Creb1^−/−^* mice die shortly after birth due to respiratory distress and show delayed differentiation of both proximal and distal airway epithelial cell populations of the lung [4], [5]. Creb1 is a member of the Creb/Atf subfamily of cAMP responsive basic region-leucine zipper (bZIP) transcription factors. The transcriptional activities of Creb1 are primarily activated by phosphorylation at the Serine 133 (Ser133) residue in response to an increase in intracellular cAMP levels. Several hormones, growth factors and cytokines have been shown to induce Ser133 phosphorylation of Creb1 via cAMP stimulation, and activate Creb1 that is normally bound as a dimer to cAMP response elements (CRE) within the promoter regions of target genes. [6], [7]. Other members of the family include activating transcription factor 1 (Atf1) and the cAMP response element modulatory protein (Crem), both of which can also heterodimerize with Creb1, and potentially provide an additional degree of diversity in gene regulation [8].

In this study, we have further investigated the potential Creb1-mediated regulation of gene targets from our microarray list which may be important for type II AEC lipid biosynthesis, an essential process required for type-II AEC surfactant production. In particular we have examined Creb1-mediated regulation of the key rate limiting lipogenic enzymes; fatty acid synthase (*Fasn*) and steroyl-CoA desaturase 1 (*Scd1*), both of which previously showed highly reduced mRNA levels in *Creb1^−/−^* fetal lungs [4]. The cytosolic Fas enzyme is a multifunctional homodimeric complex which promotes de-novo synthesis of saturated fatty acids [9], [10], while Scd1 is an endoplasmic reticulum-based transmembrane enzyme which catalyses the conversion of saturated to monounsaturated fatty acids, which then serve as substrates for synthesis of phospholipids, triacylglycerols (TAGs) and cholesteryl esters (CEs) [9], [10], [11]. Phospholipid (PC) in particular is an essential component of lung surfactant and accounts for approximately 90% of endogenous surfactant material [12]. Transcriptional and post-transcriptional regulation of both *Scd1* and *Fasn* has been studied extensively in the context of obesity and cancer development in tissues with known roles in lipogenesis such as liver and adipose tissue [11]. However little is known about the regulatory mechanisms for these factors during lung development and their potential role in fetal surfactant biosynthesis.

Therefore, in this study we hypothesized that Creb1 positively regulates gene expression of factors which may be required for type II AEC lipid biosynthesis, in particular *Fasn* and *Scd1*. We examined gene and protein expression of *Fasn* and *Scd1* using both *in vivo* and *in vitro* models where Creb1 function is either lost or inhibited, and show that in the case of *Scd1*, Creb1 is essential for normal expression in the lung. We also describe the ontogeny of gene expression for *Fasn* and *Scd1* during late respiratory development, as well as the protein localisation of these factors to epithelial cell subsets within the fetal lung. Finally, we describe the specific lipid classes which show altered levels with the inhibition of *Creb1* in a mouse lung type-II AEC-like cell line. Together our results strongly suggest that Creb1 is required for normal expression of *Scd1* during lung development.

## Materials and Methods

### Animals

*Creb1^−/−^* mice and *Creb1^−/−^Crem^−/−^* double transgenic mice were generated from *Creb1^loxp/loxp^* mice as previously described [4]. *SPC-rtTA^tg/−^* and *(TetO)_7_Cre^tg/−^* mice (Jax Laboratories, Bar Harbor, ME) were each bred to *Creb1^loxp/loxp^* mice and then maintained on a *Creb1^loxp/loxp^* homozygote background. All mouse strains were maintained on a C57Bl6 genetic background. *Creb1^−/−^* embryonic lungs were obtained from timed matings of *Creb1* ^+/−^ mice on a *Crem* ^+/+^, *Crem* ^+/−^, or *Crem^−/−^* background. Lung epithelial-specific deletion of *Creb1* in fetal mice was obtained from timed matings of *Creb1^loxp/loxp^;SPCrtTA^tg/−^* mice with *Creb1^loxp/loxp^;(TetO)_7_Cre ^tg/−^* mice, with doxycycline (600 mg/kg)(Speciality Feeds, Australia) provided in the food from E6.5 till E14.5 to cause gene recombination at the *Creb1^loxp^* locus. Fetuses exposed to doxycycline with the genotype *Creb1^loxp/loxp^;SPCrtTA^tg/−^;(TetO)_7_Cre^tg/−^* were considered mutants and are referred to as *Creb1^EpiΔ/Δ^* mice. Pregnant mothers were harvested at E17.5 by cervical dislocation. Fetuses were killed by decapitation and fetal lungs were dissected and processed for RNA, protein extraction or histological analysis. A small piece of tail was also collected for genotyping at the *Creb1, Crem, SPCrtTA^tg^* and *(TetO)_7_Cre^tg^* gene loci by PCR. All animal experimentation was approved by the School of Biomedical Sciences Animal Ethics Committee, Monash University.

### Immunohistochemistry and immunofluorescence

Mice lung samples were either fixed in optimal cutting temperature (OCT) compound or in 4% paraformaldehyde/phosphate buffered saline (PBS) solution overnight at 4°C, processed, embedded in paraffin and 5 µm thick sections cut and prepared. For immunohistochemistry antigen retrieval was performed by microwaving in 10 mM sodium citrate buffer for 20 mins followed by blocking of endogenous peroxidases using 3% H_2_O_2_ in methanol for 5 mins. Sections were then blocked in 5% goat serum for 1 hr and incubated with primary antibodies: Scd1 (#2794, Cell Signalling, Danvers, MA), Fas (#3180, Cell Signalling), ProSPC (ab3786, Chemicon, Temecula, CA) and Creb1 (#48H2, Cell Signalling) overnight at 4°C or at room temperature for 1hr. Sections were then incubated with a biotinylated goat anti-rabbit secondary antibody (Vector laboratories, Burlingame, CA). Positive staining was either detected using LSAB®2 Streptavidin-HRP (Dako, Glostrup, Denmark) and diaminobenzidine (DAB) (Dako) solutions as per manufacturer's protocol for bright field imaging, or using fluorescent-labelled secondary antibodies and/or streptavidin proteins (Life Technologies, Carlsbad, CA). Finally, sections were counterstained with nuclear stains, haematoxylin for bright field imaging and hoechst (Sigma-Aldrich, St. Louis, MO) for fluorescent imaging. Negative controls included sections treated with block buffer and secondary antibody alone.

### Cell Culture

The mouse type II pneumocyte-derived cell line MLE-15 was obtained from Dr. Jeffrey Whitsett (University of Cincinnati, USA) [13]. MLE15 cells were grown in RPMI 1640 (Gibco, Grand Island, N.Y.) supplemented with 2% fetal bovine serum, (Hyclone Laboratories, Logan, UT), insulin (5 mg/L), transferrin (10 μg/ml) and sodium selenite (3 ng/mL) (Gibco), 100 U/ml penicillin, 100 μg/ml streptomycin, 10 nM hydrocortisone, 10 nM β-estradiol, 2 mM HEPES (Sigma-Aldrich, St Louis, MO, USA) and 2 mM glutamax (Gibco). Cell cultures were maintained in a humidified 37°C incubator with 5% CO_2_/95% air. For cAMP induction studies MLE-15 cells were seeded in 6-well plates (0.4×10^6^ cells/well) and 24 hrs later treated with either forskolin (10 μM) or DMSO as vehicle control.

### Transient transfection of MLE15 Cells

MLE-15 cells were transfected using Lipofectamine 2000 (Life Technologies, USA) according to the manufacturer's protocol. Cells were transfected with constructs, an empty pRc/CMV500, a constitutively-expressing mouse Crebα, or a dominant-negative isoform of mouse Creb1 (ACreb). Cells were seeded at a density of 0.3×10^6^ cells per well in a 6 well dish in regular growth medium overnight. After 24 hrs, the medium was replaced with 2 ml of pre-warmed Opti-MEMI (Life Technologies). For transfection, the DNA-lipofectamine complex was prepared and added to the cells using the following DNA/Lipofectamine ratios: 5∶1 for pRc/CMV500 vector and ACreb constructs, 2∶1 for the Crebα construct. Cell cultures were incubated at 37°C, 5% CO_2_ for 9 hrs, then the medium was replaced with MLE-15 culture medium supplemented with 2% charcoal/dextran-treated FBS. Fifteen hours later, cells were treated either with DMSO as vehicle or forskolin (10 µm) for 8 hrs in culture.

### Isolation of RNA, RT-PCR, and qPCR

Total RNA was isolated from mouse lung and cultured cells with TRIZOL reagent (Life Technologies) as per manufacturer's protocol. Isolated RNA (1µg) was reverse-transcribed (RT) to cDNA using random hexamers and M-MLV Reverse Transcriptase, RNase H Minus, Point Mutant (Promega, Madison WI). Oligonucleotide primer pairs for qPCR analyses were designed using the web-based Primer3 software [14]. cDNAs were assayed in triplicates and the levels of 18S rRNA was used as a normalising control. Primer sequences used were (5′ to 3′): *Scd1*, forward, CTCAGCACTGGGAAAGTGAG, reverse, AGATCTCTTGGAGCATGTGG; *Fasn*, forward, AGACAGAGAAGAGCCATGGAG, reverse, TGGCCCAGAACTCCTGTA; *SrebpF1*, forward, TACTCGAGCCTGCCTTCAG, reverse, TAGATGGTGGCTGCTGAGTG; *Pparγ*, forward, ATAAAGTCCTTCCCGCTGAC, reverse, AAATGGTGATTTGTCCGTTG; *C/ebpα*, forward, GCCATGTGGTAGGAGACAGA, reverse, CAAGTTCCTTCAGCAACAGC. Cycling was performed using SYBR® GreenER™ (Life Technologies) on a Rotor-Gene™3000 (Corbett Research, Sydney, Australia). qPCR data was analysed using RotorGene 6.0 software (Corbett Research) and differential expression determined using the comparative delta-delta CT method [15].

### Protein Isolation and Western Blot Analysis

Cells were lysed in lysis buffer [60 mM Tris-HCl, pH 6.8, 1 mM EdTA, pH 8, 10 mM sodium pyrophosphate, 0.1% sodium dodecyl sulphate (SDS), 0.5% sodium deoxycholate (Sigma), 1% triton X-100 (Sigma), complete protease inhibitor and phosSTOP phosphatase inhibitor cocktail tablets (Roche Diagnostics, Castle Hill, Australia)]. Cell lysates were collected, briefly sonicated, and centrifuged at 20,817×*g* for 10 mins. Protein (30–40 µg) from each sample was then subjected to gel electrophoresis under reducing conditions and transferred to Polyvinylidene fluoride (PVDF) membranes (Millipore, Billerica, MA). Membranes were first blocked in 3% non-fat dry milk in PBST for 1hr at room temperature and then incubated overnight at 4°C with primary antibodies: Scd1 (#2794, Cell Signalling, Danvers, MA), Fas (#3180, Cell Signalling) Creb1 (#48H2, Cell Signalling), pCreb (#87G3), or β-Actin (Sigma). Following brief washes the membranes were incubated for 1hr with an appropriate horseradish peroxidase-conjugated secondary antibody. The immunoreactive proteins were visualized using chemiluminescence and relative optical densities were measured using densitometry with ImageQuantTL v.7 software (GE Healthcare, Australia). Membranes were later stripped with 0.2 M NaOH for 5 min and re-probed for β-actin as the protein loading control.

### Lipid Profiling by Mass Spectrometry

In order to assess the effect of Creb1 on downstream lipid metabolism we performed lipid profiling and examined 14 lipid classes including; bis(monoacylglycero)phosphate (BMP), phosphatidylcholine (PC), phosphatidylglycerol (PG), phosphatidylethanolamine (PE), phosphatidylinositol (PI), lysophosphatidylethanolamine (LPE), alkylphosphatidylethanolamine (PE(O)), phosphatidylethanolamine plasmalogen (PE(P)), alkylphosphatidylcholine (PC(O)), phosphatidylcholine plasmalogen (PC(P)), lysophosphatidylcholine (LysoPC), ceramide (Cer), sphingomyelin (SM) and cholesterol ester (CE). For lipid profiling studies MLE-15 cells were seeded in 6 well dishes at a density of 0.25×10^6^ cells per well. Cells were transfected with the plasmids: Crebα or co-transfected with Crebα and Empty pRc/CMV500 or Crebα and ACreb as described above. Cell cultures were incubated with transfection complex at 37°C, 5% CO_2_ for 9 hrs, and then the medium was replaced with MLE-15 culture medium supplemented with 2% charcoal/dextran treated FBS. Fifteen hours later, the cells were treated either with 10 µM forskolin or DMSO as vehicle for 24 hrs. Following treatment, cells were washed with cold PBS, scraped and cell pellets were resuspended in 100 µl Tris-NaCL, pH7. Before lipid extraction and analysis, cell lysates (100 µL) were treated with the antioxidant 20 mM butylhydroxytoluene and total protein concentration was determined using the DC protein assay (Bio-Rad).x Lipids were extracted using a single phase chloroform: methanol (2∶1) method [16] from randomised MLE-15 cell homogenates using chloroform/methanol [2∶1 (v/v)] and including internal standards: 100pmol of, bis(monoacylglycero)phosphate (BMP), phosphatidylcholine (PC) 13∶0 13∶0, phosphatidylglycerol (PG) 17∶0 17∶0, phosphatidylethanolamine (PE) 17∶0 17∶0, lysophosphatidylethanolamine (LPE) 14∶0, lysophosphatidylcholine (LysoPC) 13∶0, ceramide (Cer) 17∶0 and 200 pmol of sphingomyelin (SM) 12∶0. Lipid analysis was performed by liquid chromatography, electrospray ionization – tandem mass spectrometry using a Agilent 1200 liquid chromatography system combined with an Applied Biosystems API 4000 Q/TRAP mass spectrometer with a turbo-ion spray source (350°C) and Analyst 1.5 data system [16]. Lipid concentrations were calculated by relating the peak area of each species to the peak area of the corresponding internal standard. Total measured lipids of each class were calculated by summing the individual lipid species. Results for each lipid group were normalised to PC.

### Statistical Analysis

Statistical analyses were performed using GraphPad Prism software. Comparisons between two groups were made by a Student's *t* test. Comparisons between multiple groups were analysed by 1-way ANOVA with post hoc Tukey's test for pair wise comparisons between groups. A *p*-value of less than 0.05 was considered to be statistically significant. For lipid profiling studies statistical analysis were performed using MatLab R2011b analytical software. Comparisons between two groups were made by Student's t test and corrected, where necessary for multiple comparisons by the Benjamini-Hochberg method [17].

## Results

### Loss of *Creb1* decreases *Scd1* and *Fasn* gene expression in the fetal mouse lung

**Figure 1:**
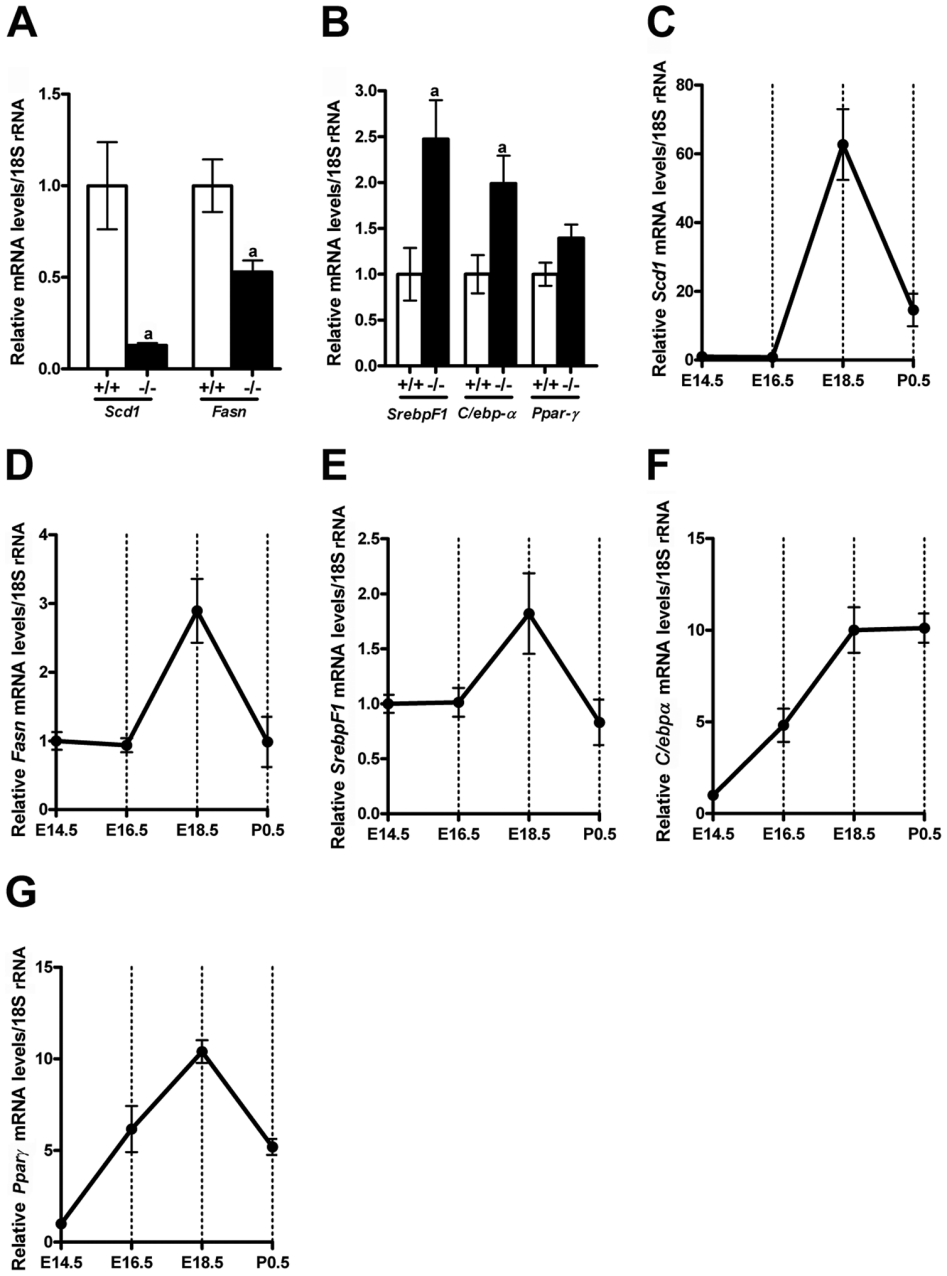
Analysis of mRNA levels by qPCR for *Scd1* and *Fasn*, and lipogenic transcription factor genes during mouse lung development. qPCR confirmed changes in *Scd1* and *Fasn* mRNA levels in the lung of E17.5 *Creb1^−/−^* fetal mice compared to littermate controls (A). Analysis of mRNA levels for the transcription factors *SrebpF1*, *C/ebpα* and *Ppparγ* in the lung of E17.5 *Creb1^−/−^* fetal mice compared to littermate controls (B). Ontogeny analysis of gene expression in normal fetal mouse lung for *Scd1* (1C), *Fasn* (1D), *SrebpF1* (1E) *C/ebpα* (1F) and *Ppparγ* (1G). All values for qPCR analysis were normalized to levels of 18SrRNA (18S). For Figs. 1C–1G the relative expression was compared with that at E14.5, which is given a value of 1. In the bar graphs, open bar – wildtype (+/+), shaded bars – E17.5 *Creb1^−/−^* (−/−) lung samples. Data is presented as the mean ± SEM, n = 4; where ‘a’ represents p-value <0.05.

Microarray analysis of the E17.5 *Creb1^−/−^* mouse lung and littermate controls identified two lipogenic gene targets: *Scd1* (7.8 fold, *p*<0.05) and *Fasn* (1.9 fold, *p*<0.05) to be significantly down-regulated in *Creb1^−/−^* fetal mice (Table 1), that was subsequently verified by qPCR (Figure 1A). Interestingly the mRNA levels of other transcription factors that regulate pulmonary lipogenesis, such as *SrebpF1*, *C/ebpα* and *Pparγ* were also analysed and found to be moderately elevated in total RNA from the *Creb1^−/−^* E17.5 lung (Figure 1B). Gene expression levels of these lipogenic genes and transcription factors were found to be developmentally regulated during normal lung development, with very low expression before E16.5, but thereafter there is a marked increase in mRNA levels until after birth (Figure 1C, D, E, F, G).

**Table 1:** Microarray analysis results for two *Creb1* gene targets, *Scd1* and *Fasn*, in total lung RNA of E17.5 *Creb1^−/−^* mice compared to wildtype litter mate controls.

Accession No	Name	Fold Change	P-value	Biological Function
NM__009127	Stearoyl-CoA Desaturase 1 (*Scd1*)	−8.6	0.0001	Fatty Acid Biosynthesis
NM_007988	Fatty Acid Synthase (*Fasn*)	−1.7	0.002	Fatty Acid Biosynthesis

### The *Creb1^−/−^* fetal mouse lung shows developmental delay in the induction of *Scd1* expression

**Figure 2:**
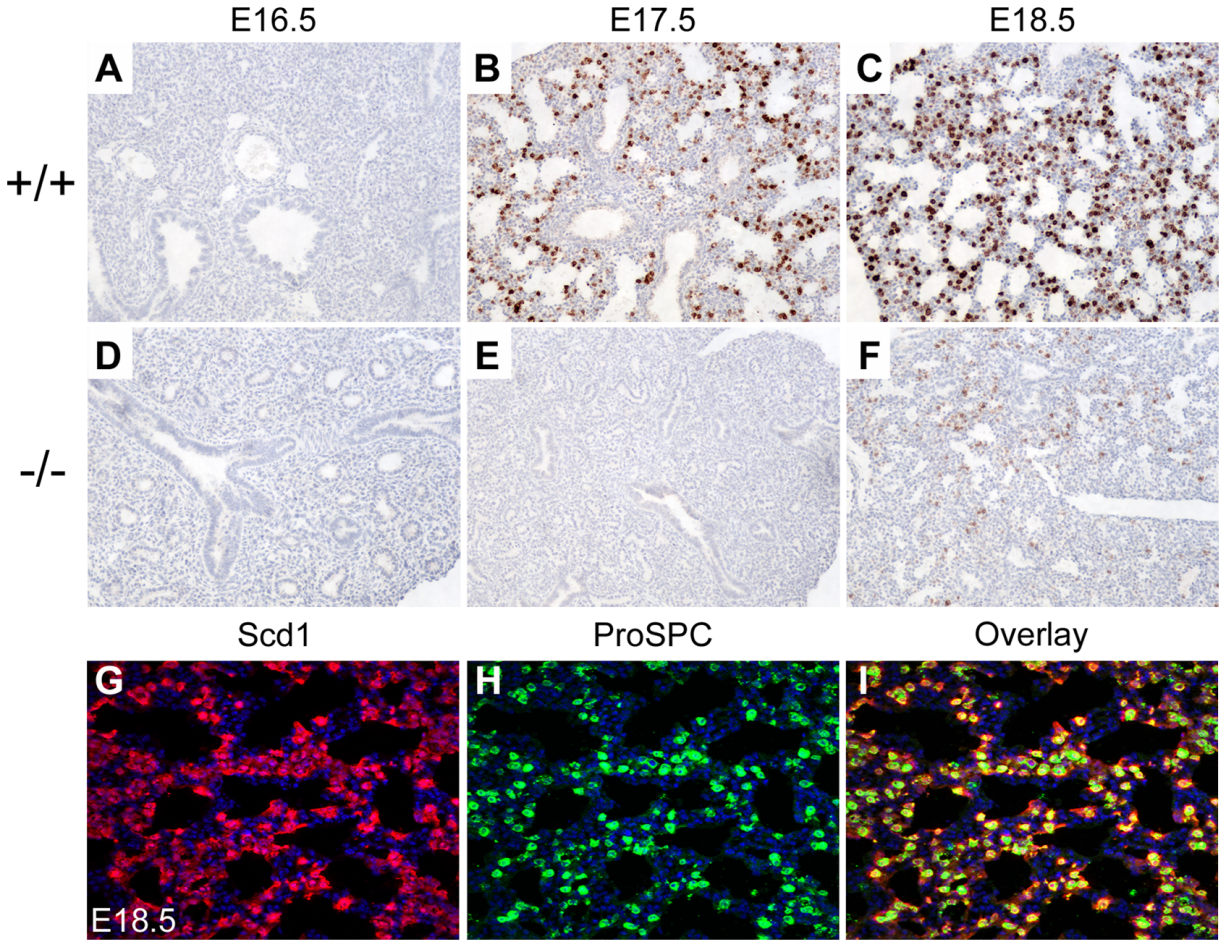
Immunohistochemical staining for Scd1 in the mouse lung from E16.5 to E18.5 in *Creb1^−/−^* mice (−/−) and wildtype litter mate controls (+/+). Staining for Scd1 was detected by E17.5 (B) in type II alveolar epithelial cells and increased at E18.5 (2A–C). In lung sections from *Creb1^−/−^* mice Scd1 was absent at E16.5 and E17.5 with very weak staining detected at E18.5 (2D–F). Scd1 staining was restricted to mature type II alveolar epithelial cells in E18.5 fetal mouse lungs and was predominantly co-localized with the type II epithelial cell marker, pro-surfactant protein C (ProSP-C) (2G–I). All bright field micrographs are taken at the magnification of 20× and fluorescent images at a magnification of 40×.

**Figure 3:**
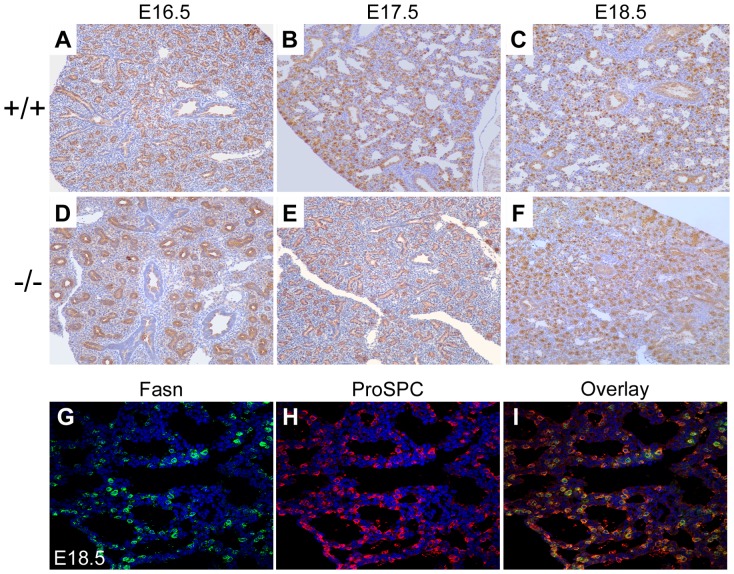
Immunohistochemical staining for Fas in the mouse lung from E16.5 to E18.5 in *Creb1^−/−^* mice (−/−) and wildtype litter mate controls (+/+). Fas protein staining was observed at E15.5 in nearly all cells in both the proximal and distal epithelial cells, and was largely restricted to type II alveolar epithelial cells at E17.5 and E18.5 (3A–C). Comparable staining was detected in lung sections from *Creb1^−/−^* mice (3D–F). Fas staining was not completely restricted to type II alveolar epithelial cells in the E18.5 fetal mouse lung when double-staining was performed with a type II epithelial cell marker, pro-surfactant protein C (ProSP-C) (3G–I). All bright field micrographs are taken at the magnification of 20× and fluorescent images at a magnification of 40×.

Immunohistochemistry of the mouse lung from E16.5 to E18.5 showed a dramatic delay in Scd1 (Figure 2A–F) protein levels in the *Creb1^−/−^* fetal mouse lung compared to wildtype controls, however we did not observe any alteration in levels of Fasn protein (Figure 3A–F). In controls, Scd1 protein was first detected at E17.5 (Figure 2B), concurrent with the normal differentiation of distal epithelial progenitors into AECs during the cannalicular to saccular stage of mouse lung development. Scd1 protein was restricted in expression to the cytoplasm of type-II AECs as shown by co-localisation with the type-II AEC marker ProSPC (Figure 2G–I). Conversely, Fas protein was detected as early as E15.5 in the lung (data not shown) and was expressed in the cytoplasm of all distal tubular epithelial cells (Figure 3A). Upon differentiation of AECs at E17.5, Fas expression similarly became restricted to type-II AECs as shown by co-localisation with ProSPC at E18.5 (Figure 3G–I), although faint expression was also observed in regions of proximal epithelium (Figure 3B & C). In the E17.5 *Creb1^−/−^* fetal lung, Scd1 protein was only detected in a few isolated cells, with a slight increase in levels at E18.5, indicative of a delay in the induction of expression (Figure 2E & F). Pulmonary Fas expression however was always comparable in staining intensity between wildtype and *Creb1^−/−^* fetal mice.

**Figure 4:**
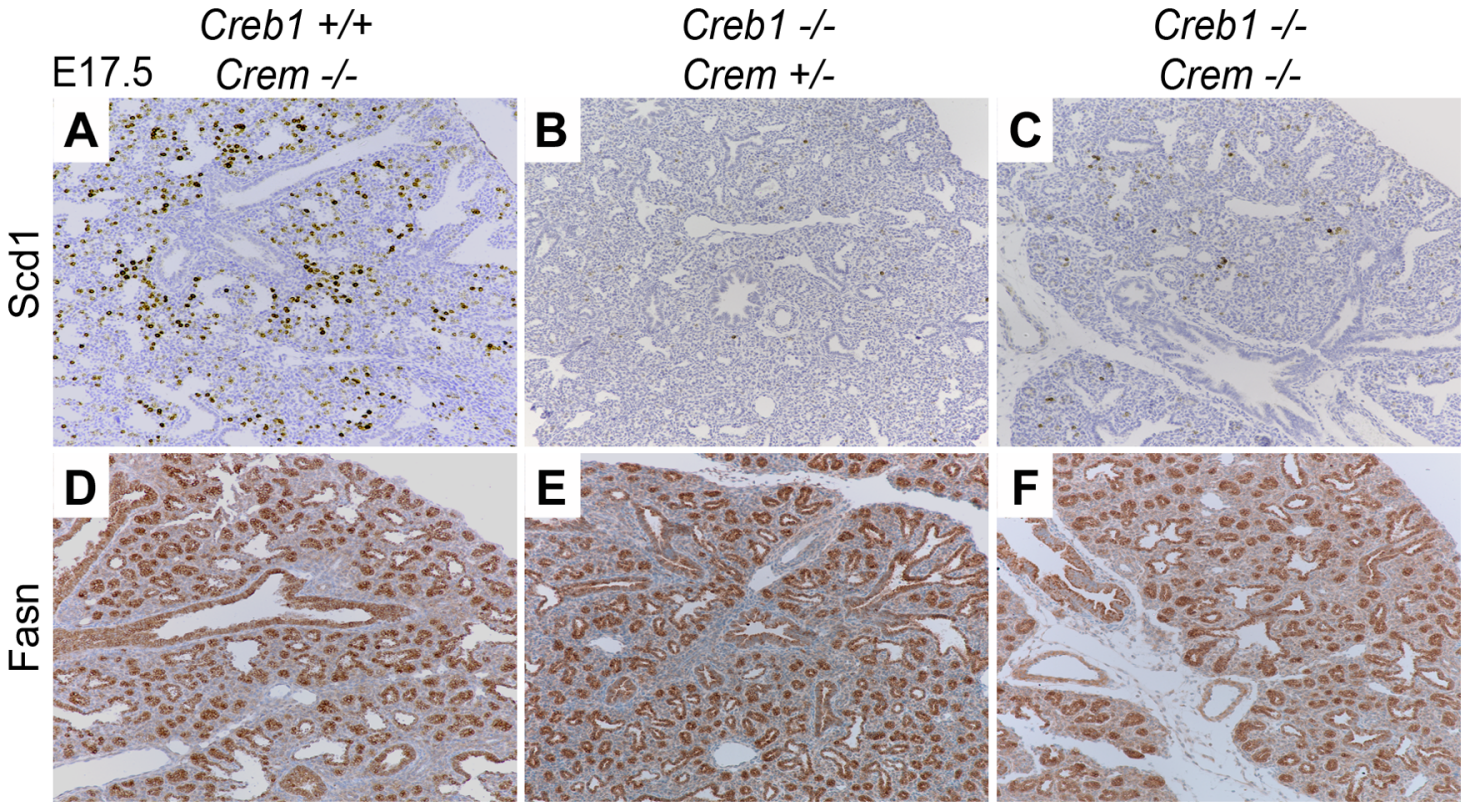
Immunohistochemical staining for Scd1 and Fas protein on E17.5 lung sections from *Creb1^−/−^* and wildtype control mice on various Crem KO genetic backgrounds. Immunostaining for Scd1 and Fas at E17.5 on a homozygous or heterozygous *Crem* genetic background showed similar Scd1 staining in lung sections from *Creb1^+/+^* and *Creb^−/−^* mice (4A–C), Fas immunostaining in *Creb1^+/+^* on *Crem^−/−^* (4D) and *Creb1^−/−^* on *Crem^+/−^* (4E) or *Crem^−/−^* (4F) genetic backgrounds were identical to that for the E17.5 *Creb1^−/−^* lung. All bright field micrographs are taken at the magnification of 20×.

Loss of *Creb1* in mice is known to cause upregulation of the related bZIP family member *Crem* in several tissues, including the fetal lung, as a mechanism to potentially compensate for loss of Creb1 function [4], [18]. To address the potential of *Crem* to compensate for the loss of *Creb1* in the lung, Scd1 and Fas protein levels were examined by immunohistochemistry in the E17.5 fetal *Creb^−/−^* mouse lung on a homozygous *Crem^−/−^* or heterozygous *Crem^+/−^* genetic background. Using immunohistochemistry Scd1 protein levels in *Creb1^+/+^* fetal lungs on a *Crem^−/−^* background were similar to E17.5 wildtype fetal mice (Figure 4A, and compared with Figure 2B). In addition Analysis of Scd1 mRNA levels in total lung RNA from E17.5 showed no statistical difference between *Crem* wildtype and *Crem^−^* ^/*−*^ fetal mice (Figure S1). Loss of *Creb1* on a *Crem^+/−^* or *Crem^−/−^* genetic background showed almost no detection of Scd1 protein identical to an E17.5 *Creb1^−/−^* fetal lung (Figure 4B & C and compare with Figure 2E). Fas protein levels on all *Crem* genetic backgrounds were also similar to the E17.5 *Creb1^−/−^* lung (Figure 4D–F, and compared with Figure 3B & E).

### Pulmonary *Scd1* expression is severely reduced in lung epithelial cell-specific *Creb1^EpiΔ/Δ^* fetal mice

**Figure 5:**
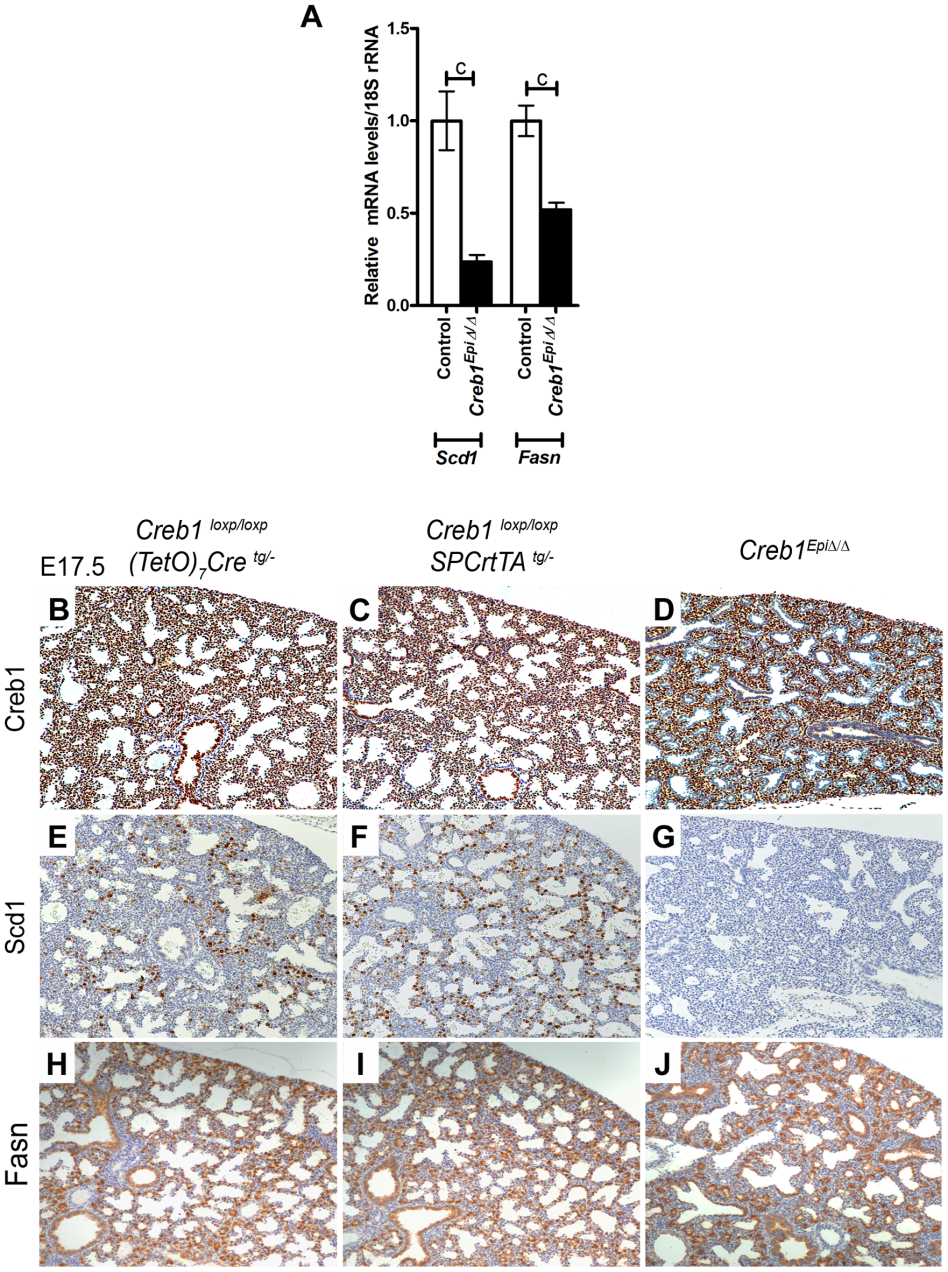
*Creb1* is required for specific activation of *Scd1* in mouse lung epithelial cells. Analysis of *Scd1* and *Fasn* mRNA levels in the lung epithelial-cell-specific *Creb1* ^Epi *Δ/Δ*^ fetal mice (A). *Creb1* immunostaining at E17.5 in lung of *Creb1* ^Epi *Δ/Δ*^ (5B–D) showed *Creb1* staining is absent in proximal and distal lung epithelial cells. Scd1 staining was absent in E17.5 *Creb1* ^Epi*Δ/Δ*^ lung sections when compared to *(TetO)_7_Cre* and *SP-C rtTA* transgenic control fetal mice (5E–G), while Fas staining was unaltered (5H–J).

Systemic loss of *Creb1* in mice produces a wide range of physiological defects including myogenic dysfunction [5], disrupted T cell differentiation, impaired brain development [19] and reduced body size [4], [19], which may indirectly influence lung development and thus confound analysis of the *Creb1^−^* ^/*−*^ respiratory phenotype. To evaluate if loss of *Creb1* specifically in lung epithelial cells would mimic our finding of disrupted Scd1 expression in the *Creb1^−^* ^/*−*^ fetal lung. Mice with a lung epithelial cell-specific *Creb1* knockout (*Creb1* ***^Epi^*** ^*Δ/Δ*^) were generated using a doxycycline-inducible Cre/LoxP approach whereby Cre recombinase was expressed under the conditional control of the human *SFTPC* promoter [20]. Using immunohistochemistry, we first tested for the presence or absence of Creb1 protein in the lung epithelial cells. E17.5 control mice showed almost ubiquitous expression of Creb1 similar to previous findings in normal fetal mouse lung of a comparable age [4] (Fig. 5B, C). In E17.5 *Creb1* ***^Epi^*** ^*Δ/Δ*^ mice Creb1 expression was almost completely absent in the lung epithelial cells (Fig. 5D). Using qPCR, we detected a statistically significant decrease of both *Scd1* and *Fasn* mRNA levels in the lung of E17.5 *Creb1* ***^Epi^*** ^*Δ/Δ*^ fetal mice relative to controls (Figure 5A). Furthermore, Scd1 immunostaining was completely absent in lung sections from E17.5 *Creb1* ***^Epi^*** ^*Δ/Δ*^ transgenic fetal mice (Figure 5G). Consistent with our previous findings, Fas protein levels remained unaltered with the loss of *Creb1* in the lung epithelial cells (Figure 5H–J).

### Scd1 expression is induced by forskolin in cultured mouse lung epithelial MLE-15 cells

**Figure 6:**
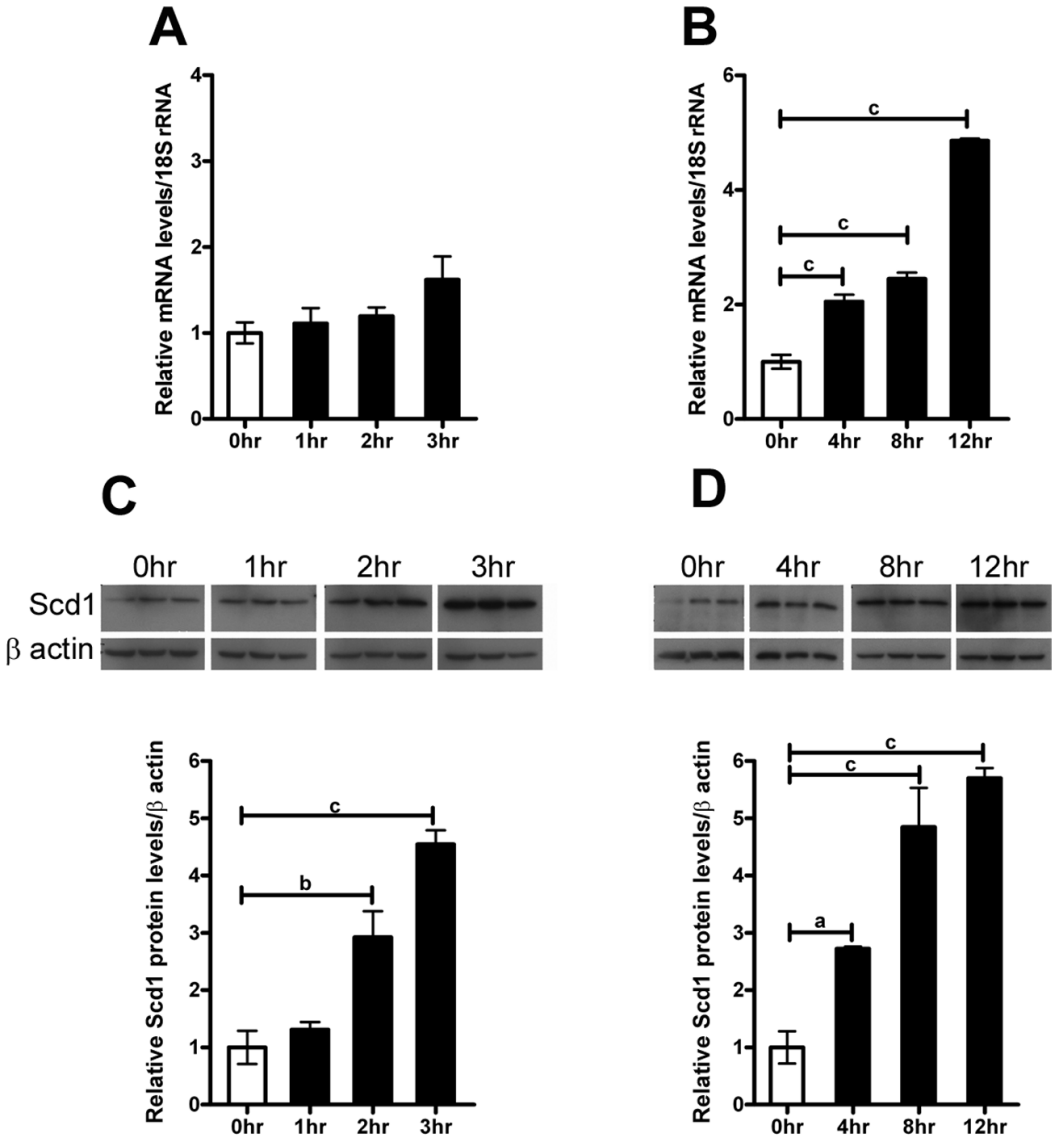
Induction of Scd1 by cAMP in MLE15 lung epithelial cells. Cultured MLE-15 cells were treated with 10 µM forskolin for various times and Scd1 mRNA levels were measured by qPCR (6A and B). For qPCR analysis all values were normalized to levels of 18SrRNA (18S). Scd1 protein levels were measured by western blot analysis and representative western blots are shown following treatment with 10 µM forskolin (6C and D). Scd1 immunoreactive bands were detected at approximately 32 kDa. Bar graphs represent relative abundance of immunoreactive Scd1 after treatment with control (open bar) or 10 µM forskolin (shaded bars) and were normalised to the level of β-actin. Data is presented as the mean ± SEM, n = 4; where ‘a’ represents p-value <0.05, ‘b’ represents p-value <0.001 and ‘c’ represents p-value <0.0001.

The induction of *Scd1* and *Fasn* was next investigated *in vitro* using cultured mouse lung epithelial MLE15 cells treated with forskolin to induce levels of intracellular cAMP. MLE15 cells showed increased levels of phosphorylated Creb1 protein after 1 hr, which returned to basal levels 3 hrs after forskolin treatment (data not shown). Creb1-mediated induction of *Scd1* levels was examined following treatment with 10 µM forskolin. Short-term treatment did not significantly induce *Scd1* mRNA levels (Figure 6A) and treatments of 4, 8 and 12 hrs (Figure 6B) were required to achieve statistically significant induction in these cells. Interestingly, treatments of 2 to 3 hrs forskolin increased *Scd1* protein levels that were sustained with 4, 8 and 12 hrs of forskolin treatment in culture (Figure 6C–D).

### Expression of dominant-negative ACreb in MLE-15 cells inhibits forskolin-mediated induction of *Scd1* gene expression

**Figure 7:**
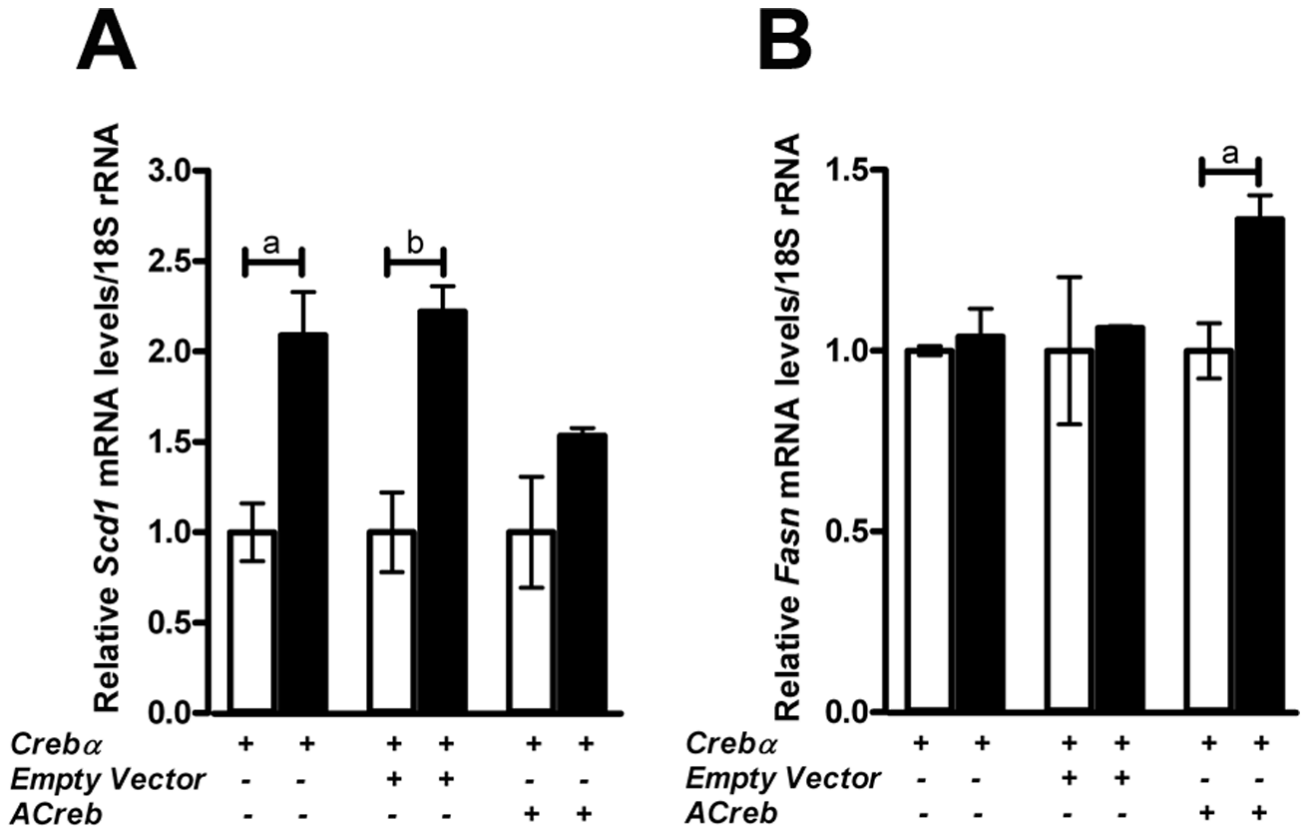
MLE15 lung epithelial cells expressing a dominant negative *ACreb* show that *Creb1* is essential for induction of *Scd1* mRNA. MLE-15 cells transfected with *Crebα* showed a 2.1 fold induction in *Scd1* mRNA following treatment with 10 µM forskolin in culture (7A). MLE15 cells that were co-transfected with *Crebα* and a dominant negative A*Creb*, showed ablated forskolin induction of *Scd1* mRNA compared to an empty plasmid control, where forskolin gave a 2.2 fold induction in *Scd1* mRNA. There was no change in *Fasn* mRNA levels with forskolin treatment but the mRNA levels were induced by the ablation of *Creb1* in MLE15 cells (7B). Data is presented as the mean ± SEM, n = 4; where ‘a’ represents p-value <0.05, ‘b’ represents p-value <0.001 and ‘c’ represents p-value <0.0001.

To determine if *Creb1* is required for the forskolin-induced increase in *Scd1* mRNA and protein levels observed previously and not via other cAMP regulated pathways, cultured MLE15 lung epithelial cells were transiently transfected with either an empty pRc/CMV500 plasmid or a plasmid that expresses a dominant-negative Creb1 isoform, ACreb. ACreb prevents Creb1-DNA binding via homodimerization with the wildtype *Creb1* leucine zipper domain [21]. To increase the sensitivity of MLE15 cells to Creb1-mediated signalling, cells were also transiently transfected with a construct which overexpresses mouse Crebα, a *Creb1* splice variant which encodes all important functional domains and is one of the most abundant *Creb1* isoforms [22]. MLE-15 cells transfected with Crebα or the empty plasmid alone showed a 2.1 and 2.2 fold induction in *Scd1* mRNA levels, respectively (*p*<0.05) following 10 µM forskolin treatment after 8 hrs in culture (Figure 7A). In cells co-transfected with Crebα and the dominant-negative ACreb, the statistically significant forskolin induction of *Scd1* mRNA levels was abolished (Figure 7A). In contrast, *Fasn* mRNA levels were not induced by forskolin treatment in MLE15 cells transfected with either the empty or Crebα plasmid alone but interestingly were increased to a statistically significant level upon co-transfection with the ACreb plasmid (*p*<0.05) (Figure 7B).

### Loss of Creb1 activity reduced ceramide, lysophosphatidylcholine, phosphatidylethanolamine and relative plasmalogen levels in MLE-15 cells

**Figure 8:**
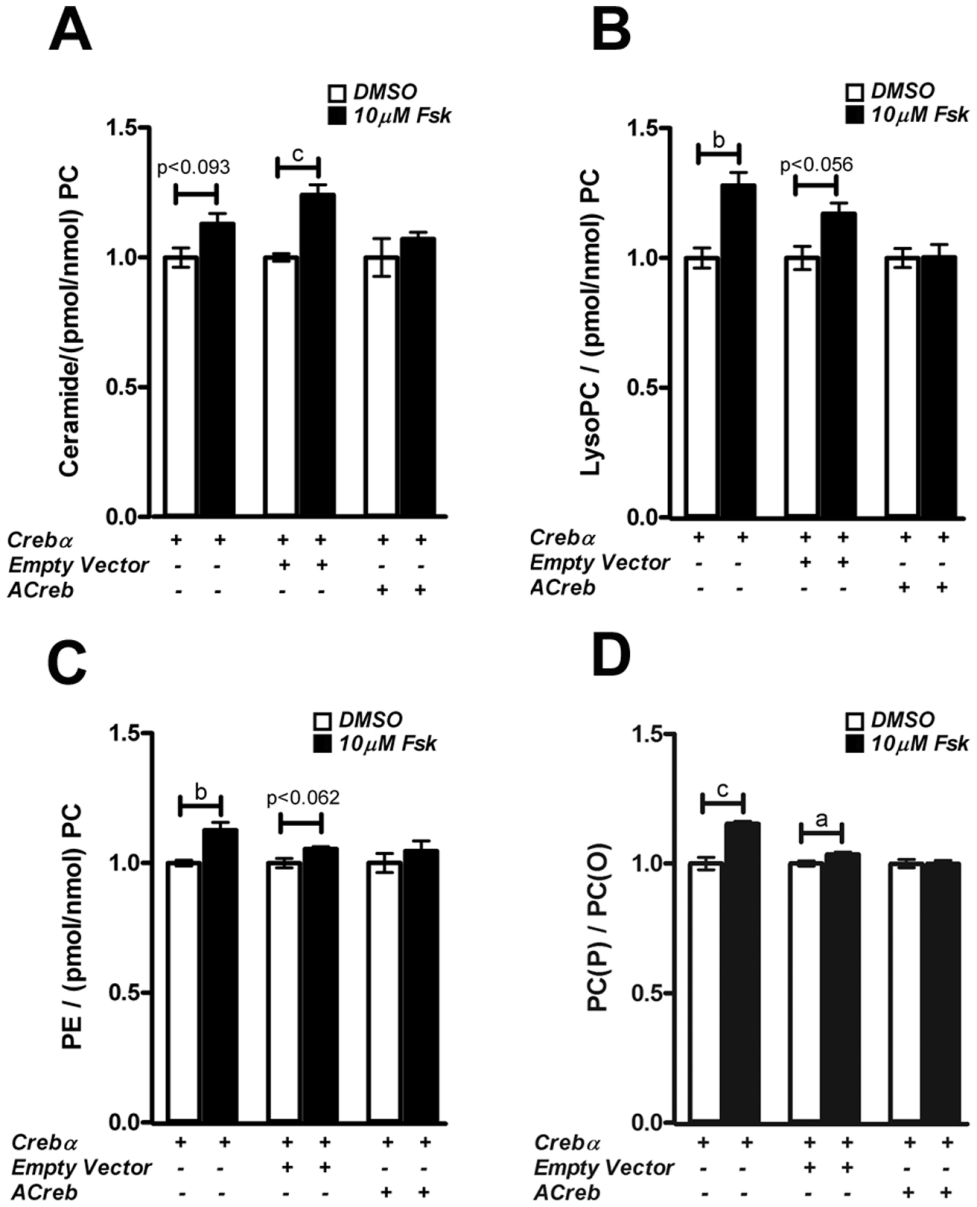
Lipidomic analysis of MLE15 cells with the loss of Creb1 activity. Inhibition of *Creb1* in ACreb-transfected MLE15 cells abolished the forskolin-induced increase in ceramide (8A), lysophosphatidylcholine (LysoPC) (8B), and phosphatidylethanolamine (PE) (8C) levels, and the ratio of alkenylphospatidylcholine (PC(P))/alkylphosphatidylcholine (PC(O)), (8D). In the bar graphs, open bars – DMSO or vehicle treated, shaded bars – 10 µM forskolin treated samples. Data is presented as the mean ± SEM, (n = 6), normalised to total phosphatidylcholine (PC); where ‘a’ represents p-value <0.05, ‘b’ represents p-value <0.001 and ‘c’ represents p-value <0.0001.

To examine the role that Creb1 plays in regulating lipogenesis and surfactant biogenesis in lung alveolar type II cells, a whole cell lipidomic analysis was performed on MLE15 cells following transient transfection with plasmids expressing the Crebα or dominant-negative ACreb1 isoforms. MLE-15 cells were transfected as described above and treated with 10 µM forskolin for 24 hrs. Whole cell lipids were extracted from treated cells and quantitated for lipid components by mass spectrometry. Inhibition of *Creb1* by transfection of the ACreb isoform in MLE15 cells abolished the forskolin-induced increase in ceramide levels (Figure 8A). Forskolin-induced increases in lysophosphatidylcholine and phosphatidylethanolamine levels were also abolished in the ACreb transfected cells (Figure 8B and C). ACreb did not abolish an increase in alkylphosphatidylcholine levels, but did reduce the forskolin-induced increase in alkenylphospatidylcholine levels (data not shown). Furthermore, the relative concentrations of alkenylphospatidylcholine/alkylphosphatidylcholine in cells transfected with the ACreb plasmid was decreased (Figure 8D). There were no changes in any other lipid species measured (data not shown). The forskolin-induced increase in the levels of lipid classes shown here were all significant before correction for multiple comparisons.

## Discussion

In this study we have demonstrated that the cAMP signalling pathway via Creb1 has an important function in pulmonary lipogenic pathways via regulation of specific lipogenic-associated genes expressed in the lung epithelial cells. Both microarray and qPCR analyses showed that loss of *Creb1* leads to markedly reduced gene expression of the lipogenic enzymes *Scd1*and *Fasn* at E17.5 indicating that these factors may be upregulated by *Creb1* in the developing lung late in gestation. We further confirmed that Fas and Scd1 proteins are localised to type-II AECs during the canalicular and saccular stages of lung development. AECs first differentiate from distal epithelial progenitors, suggesting that Fas and Scd1 have an important role within type-II AECs close to the time of birth. Given that Ser133 phosphorylation of Creb1 occurs primarily in distal lung epithelial cells, our finding of Creb1-mediated regulation of epithelial-expressed factors such as Scd1 and Fas is not surprising. Furthermore, the rapid induction of these genes between E16.5 to E18.5 occurs concurrently with the onset of the respiratory phenotype in *Creb1^−^* ^/*−*^ lungs, suggesting a temporal link between *Creb1* function and the induction of *Scd1* and *Fasn* gene expression [4]. Similar timing of *Scd1*and *Fasn* gene induction has also been noted in fetal rat lungs [23].

Our imunohistochemical data did not show any noticeable requirement for *Creb1* in regulating *Fasn* expression in the fetal lung. We suggest this may be due to a lack of sensitivity inherent with immunohistochemical staining which would not detect the relatively low magnitude of change in *Fasn* mRNA levels observed in E17.5 *Creb1^−^* ^/*−*^ lungs and the ontogeny analysis. Similarly, *Fasn* mRNA levels rise only minimally between E19 and E21 in the fetal rat lung [23]. However, *Creb1* has been shown to bind putative CREs found in the mouse *Fasn* promoter and stimulate transcription of a linked luciferase reporter gene in 3T3-L1 preadipocytes [24]. Nevertheless, our results suggest that *Creb1* may play only a minor role in promoting *Fasn* expression during late lung development. We also find *Fasn* levels are similar at E15.5, well before the observed *Creb1^−^* ^/*−*^ lung phenotype. It is also likely that levels of *Fasn* are tightly regulated by a number of transcriptional pathways, as loss of even a single *Fasn* allele in mice leads to a severe reduction in viability during embryonic development [10].

In contrast, *Creb1* function is clearly essential for *Scd1* gene expression and protein levels in the developing lung *in vivo*. Additionally our *in vitro* studies using MLE15 cells confirm that cAMP-mediated stimulation of *Scd1* expression requires *Creb1*. Arguably the bulk of literature associated with analysis of *Scd1* gene regulation has been conducted in mouse 3T3-L1 preadipocytes. In these cells cAMP analogues have been shown to stimulate *Scd1* mRNA levels [25], while knockdown of *Creb1* function using siRNA prevents the stimulation of *Scd1* mRNA levels caused by Antimycin A-mediated inhibition of mitochondrial activity [26]. However, in our studies *Scd1* mRNA was only induced to a statistically significant level after long-term forskolin exposure, indicating that Creb1-mediated regulation of *Scd1* gene expression is possibly indirect and requires other intermediary factors. In agreement with our findings, maximal cAMP-mediated induction of *Scd1* mRNA in mouse 3T3-L1 preadipocytes was also shown to require new protein synthesis [25]. Although a CRE has been found in the mouse *Scd1* promoter [25], [27], it is uncertain whether this has any biological relevance. Interestingly however we find that Scd1 protein synthesis in MLE15 cells was induced after only 2 hours of forskolin exposure, raising the possibility that *Scd1* mRNA can be stored and translated by a Creb1-dependent mechanism to enable rapid protein synthesis. While most studies find Scd1 expression is regulated primarily by gene transcription, post-transcriptional mechanisms have also been described [11], [28]. Future studies are therefore necessary to determine whether *Scd1* mRNA storage and rapid protein synthesis is a functional mechanism within type-II AECs in the developing lung *in vivo*. Although it is clear that *Scd1* is highly expressed in type-II AECs during lung development, its precise lipogenic role remains unclear. It is unlikely that *Scd1* plays a crucial role in lung surfactant function given that *Scd1^−^* ^/*−*^ mice do not appear to exhibit any respiratory phenotype [29]. However it is also possible that downregulation of *Scd1* in the lung could be compensated for by other Scd isoforms, of which four exist in the mouse genome [11]. Alternatively, Scd1 may be more involved in non-surfactant lipid biosynthesis.

The lipid profiling in MLE15 cells indicate that blockade of *Creb1* activity in lung epithelial cells leads to reduced levels of ceramide, phosphatidylethanolamine, lysophosphatidylcholine and a decreased ratio of alkenyphosphatidylcholine to alkylphosphatidylcholine. *Creb1* has been shown to induce transcription of the ceramidase gene, *ASAH1*, which is involved in the catabolism of ceramides in H295R adrenocortical cells [30]. The loss of *Scd1* in skeletal muscles of ob/ob mice is also reported to result from reduced ceramide synthesis [31], [32]. However, in the human lung epithelial A549 cell line, the reduction of Scd1 leads to reduced cell proliferation with high rate of cell death that is independent of ceramide synthesis [33].

Surfactant lipids such as lysophosphatidyl choline and phosphatidylethanolamine are present in trace amounts in the adult mouse lung and are thought to influence surfactant metabolism [34]. Creb1-mediated induction of Scd1 enzyme in the lung may be an intracellular mechanism to process any excess saturated fatty acids during lung epithelial differentiation late in development. During this stage there is a dramatic increase in *de novo* lipogenesis for surfactant biosynthesis and secretion. The increase in saturated fatty acids may also relate to the relative decrease in lysophosphatidylcholine. The polyunsaturated fatty acid species typically in the sn2 position of phosphatidylcholine are more prone to phospholipase action than saturated species, thus a decrease in the level of saturation may influence the production of lysophosphatidylcholine. Phosphatidylethanolamine is also biosynthetically related to phosphatidylcholine as a precursor which can undergo multiple methylation steps via the action of phosphatidylethanolamine *N*-methyltransferase to produce phosphatidylcholine. These results suggest that there may be a decrease in phospholipase A2 activity that would degrade both cell membranes and surfactant to increase levels of these lipid species [35]. High levels of these lipids are known to increase the sensitivity to protein inhibition that decreases surfactant bioactivity and is observed in respiratory distress syndromes and lung injury [34], [35], [36].

We did not observe any change in the levels of storage lipids such as cholesterol esters, cholesterol and triglycerides with inhibition of *Creb1*. Hence it is likely that the developing respiratory epithelial cells are preferentially glycolytic in nature rather than lipogenic. Gene deletion of SREBP cleavage-activating protein gene, *Scap* in mice have shown compensatory up-regulation of triglyceride synthesis and the accumulation of resident lipofibroblasts with the loss of lipogenesis in lung epithelial cells [37]. This also enhances type II AEC's ability to synthesize and traffic triglycerides for incorporation into disaturated phosphatidylcholine in surfactant lipids during late gestation [38]. This increase in triglyceride production by lipofibroblasts is thought to be primarily by an increase of cAMP levels that makes these cells more responsive to stimulation by other hormones such as the glucocorticoids and is driven by hormones such as prostaglandin PGE_2_ late in gestation [38]. The findings of this study suggest that Creb1-mediated cAMP signalling plays a role in the maintenance of surfactant homeostasis and bioactivity via regulation of lipid classes in mesenchymal lung cell populations such as lipofibroblasts. In summary, both *in vivo* and *in vitro* studies presented here demonstrate that *Creb1* is an essential transcription factor regulating the induction and levels of *Scd1* in type II alveolar epithelial cells during lung development.

## Supporting Information

Figure S1 Analysis of *Scd1* mRNA levels in the *Crem* ^−/−^ and *wild type* fetal mouse lung at E17.5 by qPCR. All values were normalized to levels of 18S rRNA and relative expression was compared with that of *Crem* wildtype (+/+) which was given a value of 1. In the bar graph, open bar – wildtype (+/+), shaded bar – E17.5 *Crem1^−/−^* (−/−). Data is presented as the mean ± SEM, n = 4. *p* = 0.683 and was not considered significant.
(TIF)
