# Supplementary figures and images for: cAMP Response Element Binding Protein1 Is Essential for Activation of Steroyl Co-Enzyme A Desaturase 1 (Scd1) in Mouse Lung Type II Epithelial Cells
Source: PLoS One. 2013 Apr 18;8(4):e59763. doi: 10.1371/journal.pone.0059763 (PMC3630165; doi:10.1371/journal.pone.0059763)

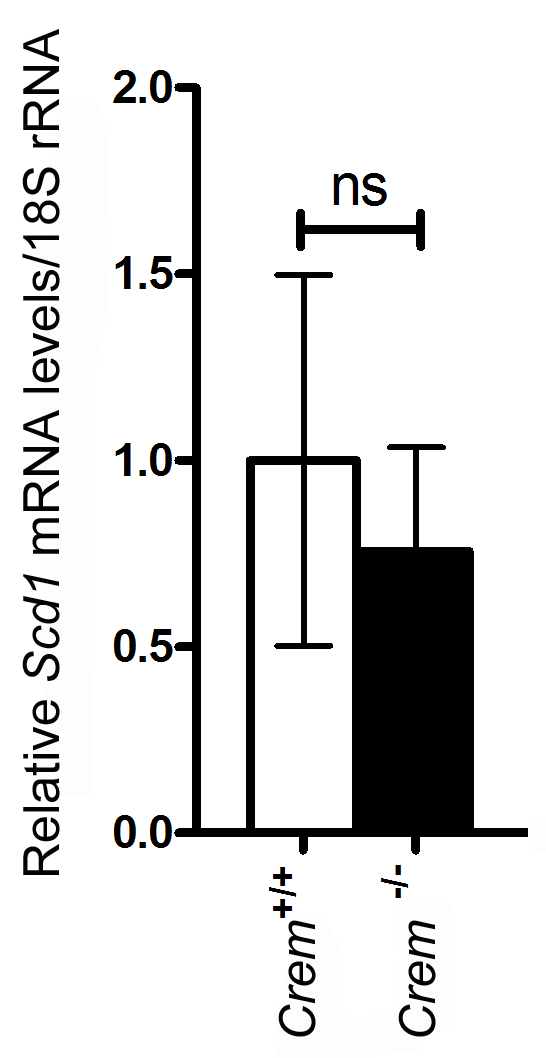

Supplement: Figure S1 — Analysis of Scd1 mRNA levels in the Crem −/− and wild type fetal mouse lung at E17.5 by qPCR. All values were normalized to levels of 18S rRNA and relative expression was compared with that of Crem wildtype (+/+) which was given a value of 1. In the bar graph, open bar – wildtype (+/+), shaded bar – E17.5 Crem1−/− (−/−). Data is presented as the mean ± SEM, n = 4. p = 0.683 and was not considered significant. (TIF) [file pone.0059763.s001.tif]
